# Initial single surgeon evaluation comparing C-arm fluoroscopy with the Cirq robotic assistance device for instrumentation of the thoracolumbar spine

**DOI:** 10.1186/s12893-022-01878-4

**Published:** 2022-12-19

**Authors:** Sohum K. Desai, Jennifer P. Adams

**Affiliations:** 1 Department of Surgery, University of Texas Rio Grande Valley School of Medicine, Harlingen, TX USA; 2 University of Texas Rio Grande Valley School of Medicine, Harlingen, TX USA

**Keywords:** Brainlab, Cirq, Thoracolumbar

## Abstract

**Objective:**

To compare our experience with pedicle screw insertion of the thoracolumbar spine utilizing the Cirq robot assistance device compared with traditional paradigm using fluoroscopy.

**Methods:**

We prospectively collected data of patients undergoing pedicle screw instrumentation in the thoracolumbar spine performed by a single surgeon at three different centers. One center took delivery of the Cirq robotic assistance device. Remaining two centers used C-arm fluoroscopy. Demographic information, diagnosis, total OR time, intraoperative complications, unexpected return to the operating room, and hospital readmissions within 90 days was compared between the two cohorts.

**Results:**

A total of 166 screws were placed during the study period. Forty percent were placed using the Cirq. Two thirds the patients had traumatic diagnoses with remaining degenerative spine disease. There were no misplaced pedicle screws in either group. While total OR time was longer in the Cirq cohort by 123 min (p = 0.04), actual procedural time was not statistically different (p = 0.11). Nonetheless there were also more hospital readmissions in the Cirq cohort compared with the C arm group (p = 0.04).

**Conclusions:**

Thoracolumbar screws inserted using C-arm fluoroscopy utilize less total operating room time with similar accuracy compared with the Cirq robotic assistance device. Further studies are warranted.

## Introduction

Spinal robotic surgery is being adopted in the United States with greater frequency even outside tertiary care academic centers at the community level. Coordination of robotic surgical navigation with intraoperative computerized tomography (iCT) has improved the positional accuracy of pedicle screw placement reduced operating room staff exposure to ionizing radiation and may reduce physician musculoskeletal fatigue that could occur with multilevel constructs [1]. There are currently eight robotic systems approved in the United States by the FDA for spine surgery at the time this manuscript was written including: four iterations of Mazor (Mazor Robotics Inc, Caesarea, Israel), two of the ROSA (Zimmer Biomet, Warsaw, Indiana), ExcelsiusGPS (Globus Medical Inc, Audubon, Pennsylvania), and the Brainlab Cirq (Munich, Germany). The Cirq received FDA clearance in September 2019 however literature regarding its use still remains limited [2–4] in comparison with other available systems. To our knowledge, this is the first single-center study to compare the use of the CIRQ^®^ Robotic Alignment to standard C-arm fluoroscopy-guided pedicle screw placement during instrumentation of the thoracolumbar spine.

## Methods

A consecutive series of instrumented thoracolumbar spine cases was queried from a prospectively maintained database of a single surgeon cases (SKD). The database is compiled from three hospitals which have an IRB approved process for data collection with Metrowest Medical Center. One of the centers took delivery of the Brainlab Cirq in February 2021 [5]. The remaining centers have C-arm fluoroscopy.

The Cirq is a bed-mounted, passive robotic arm to help stabilize a drill guide. Surgery time is defined as the time period between first incision and closure. The time required for robot installation and positioning and is defined as the time necessary to position and prepare the patient and install and configure the robotic arm. Our typical robotic workflow after exposure and image acquisition consisted of positioning the drill guide into a desired trajectory based on axial and sagittal views displayed on the navigation. We then used a 3.2 mm bit to drill to a preplanned depth corresponding to 80% of the anterior–posterior width of the vertebral body. A K-wire was then placed, the drill guide removed, and an appropriately sized tap of 4.4 mm diameter was used. Finally, a cannulated modular pedicle screw from Precision Spine (Parsippany, NJ, USA) was placed. After all instrumentation was placed, a final intraoperative CT was obtained after placement of a tulip heads but before the rods and set screws were secured. Average procedure time using the Cirq was 281.75 min.

Our fluoroscopy workflow was as follows. Preoperative imaging was studied to measure the pedicle width and length for instrumentation. After prepping and draping the patient, a radio-opaque vascular tape was used to identify the instrumented levels by counting up from the sacrum with fluoroscopy. The levels were marked on the skin and we would proceed with subperiosteal exposure of the spine. We would again confirm the levels to be instrumented after exposure with fluoroscopy. Next a screw entry point was identified using anatomic landmarks. A pilot hole was created with a matchstick burr. A Lenke probe was then advanced to a depth of 40 mm. A ball tipped probe was then used to sound the pedicle for any breeches. The pedicle was then tapped and an appropriately sized screw was advanced under AP and lateral C arm fluoroscopy. Once all planned levels were instrumented, we performed EMG screw stimulation prior to placement of rods and set screws. Both methods were minimally invasive procedures.

All patients participating in this series provided their consent for the procedure as well as for the technology to be used. Demographic information, diagnosis, total OR time, procedural time, intraoperative complications, hospital re-admissions within 90 days, and unexpected return to the OR within 90 days was collected. We define total OR time as the time from when general anesthesia was started until extubation. Statistical analysis was performed using Microsoft Excel with an unequal variance T-test. The differences were considered to be significant if the statistical P-value was < 0.05.

## Results

A total of 66 screws were placed during the study period. There were 10 patients in both the robotic arm group and the fluoroscopy group. 40% were placed using the Cirq. Two thirds of the diagnoses were for trauma remaining for degenerative pathologies. Average age was 46 years old (24 to 74) in the C-arm group and 63 years old (51 to 71) in the robotic group (p = 0.13). There was no difference in actual procedural time (p = 0.11), however total OR time was longer in the robotic group by 123 min (p = 0.04) (Table 1). There were no misplaced pedicle screws in either group. Intraoperative fluoroscopy was used for the robot-guided implantation of K-wires into the pedicles and determination of proper placement of screws. After screw placement, postoperative CT scan was performed to confirm correct screw placement. There were no intraoperative complications such as spinal fluid leak in either group. There was a statistically significant increase in hospital readmissions with the Cirq cohort (p = 0.04) (Table 1). Reasons for readmission included urinary retention, severe pain or wound drainage. There were no hospital readmissions or unexpected return to the operating room in the C-arm fluoroscopy cohort. Long-term consequences, including recovery of physical function and maintenance of spinal stability were assessed by outpatient clinic visits and there were no significant reports. There were no reports of spinal instability as viewed from postoperative imaging. There was one return to the operating room in the Cirq cohort which we discuss in the vignette below and contrast with a similar case in the C arm cohort.

**Table 1:** Baseline clinical and peri-operative characteristics of patients in the C-arm and robotic group

	C-arm	Brainlab Cirq	p-value
Number of patients	18	10	–
Age (Mean)	46	63	0.13
Sex (%)	83% Male 17% Female	100% Male 0% Female	–
Construct size (Mean number of pedicle screws)	7.5	6.5	0.36
Construct size (Median)	4	4	–
Mean OR time (min)	255.5	378.5	0.04
Mean procedural time (min)	199.5	281.75	0.11
Hospital readmission, 90 days	0	3	0.03
Unexpected return to OR, 90 days	0	1	0.20

### Illustrative cases

#### Patient A

**Fig. 1:**
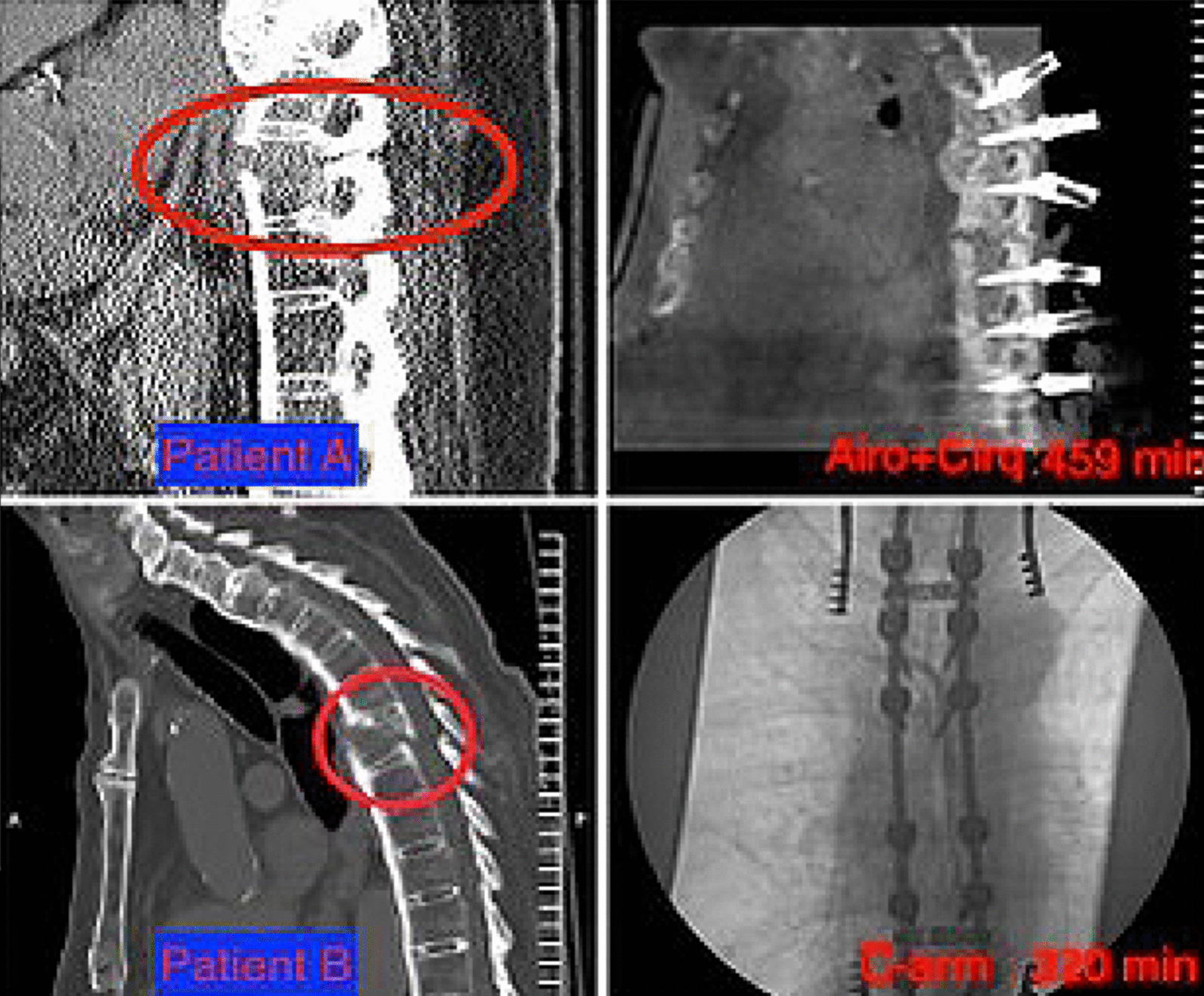
Top left demonstrates a sagittal noncontrast CT of the thoracic spine optimized for bone windows demonstrated a three-column fracture at T9 level in Patient A. The top right shows a sagittal intraoperative CT of the thoracic spine demonstrating instrumentation above and below the chance fracture. The total OR time for this particular case is noted. The bottom left displays a sagittal CT of the thoracic spine demonstrating a similar three-column fracture at T7 in Patient B. The bottom right illustrates AP image from C-arm fluoroscopy after hardware is placed. The total OR time for this case is also noted

72-year-old male with history of ankylosing spondylitis who presented to the emergency department complaining of severe back pain after a ground level fall. On physical exam he moved all extremities, normal reflexes, and intact sensation to light touch throughout. He underwent CT that showed an acute three column fracture through T9 (Fig. 1). MRI did not demonstrate any acute epidural hematoma. He underwent posterior instrumentation and posteriolateral arthrodesis from T6 to T12 utilizing the Brainlab Cirq (Fig. 1). Post operatively he was mobilized and discharged 4 days. He returned on post-operative day 17 with wound drainage. He underwent an MRI which demonstrated a complex fluid collection suspicious for suprafascial wound abscess. He underwent culture, washout, and placement of wound vacuum system. Instrumentation was left in place as the abscess was above the fascia and the recent arthrodesis had not fused yet. His culture returned positive for MRSA and the patient was placed on intravenous antibiotics for 6 weeks. At follow up at 12 weeks he had normalization of his white count and inflammatory markers. His wound had healed.

#### Patient B

74-year-old male with history of ankylosing spondylitis who presented to the emergency department who was found down by his neighbor complaining severe back pain after a ground level fall. On physical exam he was neurologically intact. He underwent CT that showed an acute three column fracture through T7 (Fig. 1). MRI also did not demonstrate any acute epidural hematoma. He underwent posterior instrumentation and posterolateral arthrodesis from T4 to T10 utilizing the C-arm fluoroscopy (Fig. 1). Post operatively he was mobilized and eventually discharged on hospital day number 4. He followed up on an outpatient basis at 2 weeks, 1 month, and 3 months without any complaints or complications.

## Discussion

To date, there are very few studies reporting results of Cirq Robotic arm in spine instrumentation. One case report of a patient with type II odontoid fracture underwent a C1–C2 posterior percutaneous fixation using Cirq Robotic Assistance coupled to the AIRO intraoperative computed tomography (iCT)-scan and BrainLab navigation system had an uncomplicated postoperative course [3]. Four screws were placed, with all of them rated as acceptable (100%).

Another case series reported seven patients undergoing posterior percutaneous pedicle fixation using Cirq robotic assistance coupled to intraoperative computed tomography scan and Brainlab navigation system. 28 screws were placed within cervical and upper thoracic pedicles, 85.7% were rated as acceptable and 14.3% as poor, according to the Neo and Heary classification. The radiation dose received by the patient was 9.1 mSv.2 and postoperative results were excellent [6]. We offer a contrasting study demonstrating longer time under anesthesia with use of the Cirq, potentially contributing to poor postoperative outcome.

Time is an important metric in the operating room. We define total OR time in this study as the total time spent under general anesthesia which is different than procedural time which begins from skin incision to wound closure. Our study observed longer total OR time by approximately 2 h even between similar sized constructs. The first results did not demonstrate a difference in actual procedural time between fluoroscopy and robotic cohorts (p = 0.11). Neither group experienced intraoperative complications such as a CSF leak or misplaced pedicle screws. Therefore, we hypothesize increased total OR time occurred due to initial Cirq preparation, including mounting the Cirq robot to an ideal position, positioning patient after obtaining general endotracheal anesthesia, and acquiring images after exposure. Additional time is related to the learning curve of the operating team, including surgeons, nurses, those tasked with surgical positioning, and company representatives for guiding the timing of installation of the robotic system. There is a possibility that different local hospital customs influenced our results and is a limitation to take note of.

We also saw a corresponding increase in hospital readmission which was statistically significant. The reasons for readmission in our series included uncontrolled pain, post-operative urinary retention, and wound drainage. Postoperative urinary retention (POUR) is a common complication after spinal surgery. Intra- and postoperative factors including operating time, anesthesia time, number of fusion levels, mobilization status prior may have an association with POUR. Patient-related factors, including pre-operative mobility status may also be associated with the likely development of POUR. The likely rationale for urinary retention as a readmission in the Cirq cohort is the longer anesthesia time [7]. This finding appears to lend credibility to our hypothesis that increased readmissions resulted from effect of prolonged anesthesia time rather than directly from surgery. Patient A in the above vignette demonstrates surgical site infection (SSI), which remains a problematic complication in the modern era of advanced operative techniques and improved perioperative care. An institutional study by Deng et al. with 2252 patients over 4-year span undergoing thoracolumbar spine surgery found that older age, ASA classification > II and longer operative times were associated with increased incidence of SSI, with the most common causative organisms being methicillin-sensitive *Staphylococcus* *aureus* and methicillin-resistant *S. aureus*. Other predictors of increased odds of SSI include Coronary artery disease, diabetes mellitus and male sex [8].

Surgical site infection relates to risk factors, including high BMI, longer operation times, diabetes, smoking, history of previous SSI and type of surgical procedure. Puffer et al. found that estimated blood loss over 1 L, previous SSI and diabetes were found to be independent statistically significant risk factors for SSI with obesity increasing the specific risk of superficial infection. This group noted that in spine surgery for deformity and degenerative disease, SSI has been associated with operative time, showing a nearly tenfold increase in SSI rates in prolonged surgery [9]. We believe that length of time in the operative room prior to surgical incision (anesthesia ready time) may be related to the risk of SSI and other complications. Radcliff et al. found that preoperative in-room time prior to the start of surgical incision is an independent risk factor for SSI [9]. Patient-related factors such as diabetes and BMI were not included in the discussion on readmissions in this report as our patients underwent appropriate preoperative screening to minimize and medically control such risk factors. Given these studies, all possible steps should be taken prior to entrance into operating room to reduce in-room time and opening of surgical sterile instrumentation should be delayed until the surgery is ready to proceed.

In contrast with that of the larger floor-mounted robotic platforms designed to maximize rigidity of the end effector of the robotic arm, the Cirq robotic assistance has a lighter design and a bedside mount. From a technical standpoint, the bed mounted feature limits the range achieved by the working arc of the Cirq. The arm must be mounted to an ideal position prior to incision where it can sufficiently reach the planned levels of surgery. As such if the device is placed too caudal it may not be able to reach the cranial most level without repositioning. Additionally we have found that contralateral cranial or caudal most screw can be difficult to triangulate due to the limited reach of the arm. The mounted design reduces the rigidity of robot arm relative to the patient and may induce a higher rate of screw misplacement. While we did not experience this issue, a study from Ringel et al. found the conventional freehand technique to have superior accuracy compared with the SpineAssist robot technique, with 93% and 85% accuracy rate for screw placement, respectively [10]. The decreased accuracy was attributed to the use of a bed-mounted frame that is not rigidly fixed to the patients’ spines.

Our findings should not be seen as an indictment against all spinal robotics or navigation systems. Spinal robotic systems have gone through an evolution process, for example, Mazor with the initial SpineAssist, Renaissance, Mazor X, and current Mazor X Stealth Edition. Future iterations of the Brainlab system such as the robotic alignment module and planning software may improve on the pitfalls of the current system.

The authors also acknowledge several limitations to this study. It represents a single surgeon experience. Further, the study is small and done on a somewhat homogenous population consisting of male trauma patients. Additionally, a learning curve exists with the adoption of any new technology. Others have demonstrated that this appears to be overcome by the placement of approximately 30 screws robotically [11]. In our case we did not see a statistically significant difference in procedural time between fluoroscopy and robotic procedures thus believe a surgeon learning curve may have less impact on our results. We acknowledge that we have not reached the end of the learning curve and the high initial time associated with use of robotic technology will continue to have implications in operating-room utilization time. The significant finding however was that pre procedural positioning and setup added to time under general anesthesia which was associated in more readmissions.

## Conclusions

The first results show that thoracolumbar screws inserted using C-Arm fluoroscopy utilize less total operating room time with similar accuracy compared with the Cirq robotic assistance device. Further studies are warranted.

## Previous presentations

A portion of the data found in this manuscript was submitted in abstract form to the Congress of Neurological Surgeons Annual Meeting October 2021 in Austin, Texas.

## Data Availability

The dataset(s) supporting the conclusions of this article is available in the MetroWest Medical Center data repository upon request.
